# Optimized platform medium and feed for rCHO cell lines using the CHEF1^® ^expression system

**DOI:** 10.1186/1753-6561-7-S6-P98

**Published:** 2013-12-04

**Authors:** William Paul, Raymond Davis, Andrew Campbell, Sarah Terkildsen, Vann Brasher, James Powell, Blake Engelbert, Howard Clarke

**Affiliations:** 1 Life Technologies Corporation (PD-Direct® Bioprocess Services), 3175 Staley Road, Grand Island, NY, 14072 USA; 2 CMC Biologics, 22021 20th Avenue SE, Bothell, WA, 98021 USA

## 

Chinese Hamster Ovary (CHO) cells are widely used in biomanufacturing and biomedical research to produce proteins of clinical significance. The environment the cells grow in to produce these proteins is complex and varies across the industry. One key variable in production processes is the cell culture medium used. Media can include chemically-defined components such as amino acids, vitamins, lipids, metal salts, and buffers. In addition, undefined components such as proteins, serum, or hydrolysates may be added. To reduce complexity, increase consistency, and comply with increasing demands from regulatory entities, chemically-defined formulations are preferred and can be developed and optimized for a given cell line. While a medium and feed can be optimized for every cell line/clone, developing a platform system provides a cost-effective option while ensuring a high level of growth and productivity.

In this collaboration, between Life Technologies PD-Direct^® ^and CMC Biologics, a single animal origin-free, hydrolysate-free base platform medium and three synergistic feed media were developed for use with recombinant CHO cell lines engineered using the CHEF1^® ^expression system to produce monoclonal antibodies. The CHEF1 expression system utilizes regulatory domains from the Chinese hamster elongation factor 1 (EF1α) gene to drive production of heterologous proteins [1]. Serum-free, suspension adapted CHO DG44 cells were transfected with CHEF1 plasmids harboring 2 different IgG1 MAb genes and used as test cell lines to develop a platform feed system. A cell culture production platform system (CHEF1, base medium, feed media) was developed and optimized using two cell lines that were previously grown in an undefined culture system. The new platform growth system developed here, showed an average 1.6 fold improvement in titer for the two cell lines compared to the performance using the undefined culture system.

Using Design of Experiment (DOE) methods, we performed a Feed Mixtures experiment and a 2-Level Categoric experiment in shake flasks (culture parameters are shown in Table 1). Cell counts and viabilities were determined using a Cedex AS20 automated cell counter (Innovatis Inc.). Product titer was measured by Protein A HPLC. Performance data from the Feed Mixtures experiment were analyzed using Design Expert^® ^(Stat-Ease^®^). Select spent media samples from the best performing Feed Mixtures conditions were analyzed for glucose, amino acids and select water-soluble vitamins using immobilized enzyme (YSI Life Sciences), UPLC (Waters AccQ-Tag™ - reverse phase with UV detection) and HPLC (ion-pair reverse phase using a UV detection), respectively. The Feed mixtures data were used to calculate nutrient consumption rates, which in turn were used to develop 3 balanced feeds (at neutral pH). A separate Feed Supplement (at high pH) was designed to facilitate delivery of components that were needed at levels above solubility limits in a neutral solution. These feeds and the Feed Supplement were then tested in a 2-Level Categoric experiment, evaluating feed volume, feed schedule, and the feed supplement. Performance data from this experiment were analyzed using Design Expert. Select spent media samples from the best performing conditions were analyzed for glucose, amino acids and select water-soluble vitamins. These data demonstrated that the three feeds were balanced and, when the feed supplement was included, provided nutrients at levels sufficient for continued growth/productivity. The best performing feed system (balanced feed [BF1] and feed supplement [FS]) was used in a bioreactor confirmatory experiment (culture parameters shown in Table 1). In addition, a day 0 feed was designed (BF5 - included recombinant growth factors) and tested in the bioreactor.

**Table 1:** Culture Parameter Conditions and Set-Points

Parameters		
**Shake Flask/Culture Volume**	125 mL vented Erlenmeyer	30 mL working volume

**Bioreactor/Culture Volume**	3 L single-use CellReady bioreactor	2 L working volume

**Seeding Density**	5x10^5 ^viable cells/ mL	

**Temperature**	37°C ± 0.5°C (days 0 - 4)	34°C ± 0.5°C (day 5 - end)

**CO_2_ Level (Shake Flask)**	6% ± 1% (days 0 - 4)	2% ± 1% (day 5 - end)

**pH (Bioreactor)**	7.0 ± 0.2	

**RPM (Shake Flask)**	120 ± 5	

**RPM (Bioreactor)**	200 ± 10	

**Dissolved Oxygen (Bioreactor)**	60% ± 5%	

Supplementing BF5 at 3% (v/v) prior to inoculation and feeding 4%BF1 on day 4, 5% on day 6, 3% on day 8, 2% on day 10, and 1% (v/v) on days 12 and 14 and FS at 0.2% (v/v) on alternate days starting on day 3 provided an environment for both cell lines that resulted in productivity superior to the control condition; cell line #1 reached 1.1 gm/L (control = 0.5 gm/L) and cell line #2 reached 2.0 gm/L (control = 1.6 gm/L).

**Figure 1:**
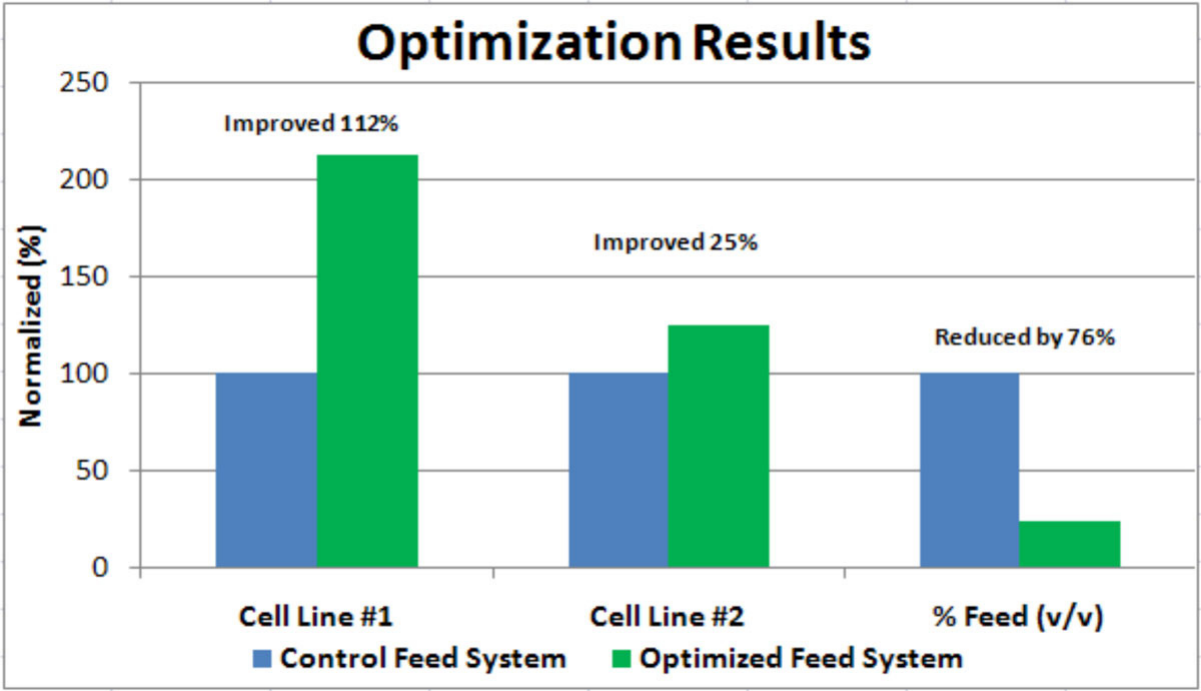
**Summary of Optimization Collaboration - Improved titer by 112% (cell line #1), by 25% (cell line #2), and reduced volume fed by 76%**.

Since the cost of dry format media is more economical than liquid media at GMP scale, the feeds were converted from liquid format to dry formats; BF1 was converted to Advanced Granulated Technology™ (AGT™) format and the Feed Supplement was converted to a dry powder media (DPM). Once hydrated, these feeds were tested to confirm equivalency, achieving similar growth and productivity patterns as their liquid counterparts. Additionally, these feeds have been concentrated to reduce the dilution effect that many commercial feeds produce, resulting in an approximate 76% reduction in feed volume added over the life of the culture (Figure 1). This is a significant reduction in the volume of fluid requiring in-process handling and downstream processing; saving time, equipment, and money. This feed system development collaboration yielded a 112% improvement (over the control condition) in product titer for cell line #1 and a 25% improvement in product titer for cell line #2 (Figure 1). The base medium and the newly developed final feed system provide an animal origin-free, hydrolysate-free growth environment. For the purposes of many commercial cGMP processes, this culture system provides an economical solution. Addition of a proprietary undefined feed (CMC Biologics), prior to inoculation, on top of this balanced feed system has been shown to boost productivity by about 15% over using just the balanced feed system. Next steps include evaluating protein quality and validating at production scale.
